# Expert Consensus on Dipeptidyl Peptidase-4 Inhibitor-Based Therapies in the Modern Era of Type 2 Diabetes Mellitus Management in India

**DOI:** 10.7759/cureus.61766

**Published:** 2024-06-05

**Authors:** Sanjay Kalra, Saptarshi Bhattacharya, A Dhingra, Sambit Das, Nitin Kapoor, Shehla Shaikh, Vivek Kolapkar, R V Lokesh Kumar, Kamlesh Patel, Rahul Kotwal

**Affiliations:** 1 Endocrinology, Bharti Hospital, Karnal, IND; 2 Endocrinology, Indraprastha Apollo Hospital, Delhi, IND; 3 Endocrinology, Ganganagar Super Speciality Clinic, Ganganagar, IND; 4 Endocrinology, Kalinga Institute of Medical Sciences, Bhubaneswar, IND; 5 Endocrinology, Diabetes and Metabolism, Christian Medical College and Hospital, Vellore, IND; 6 Endocrinology, Saifee Hospital, Mumbai, IND; 7 Medical Affairs, Lupin Limited, Mumbai, IND

**Keywords:** vildagliptin, type 2 diabetes mellitus, individualized therapy, glycemic control, dpp-4 inhibitors, combination therapy

## Abstract

India has a high prevalence of type 2 diabetes mellitus (T2DM) with unique clinical characteristics compared to other populations. Despite advancements in diabetes therapy, a significant number of patients in India still experience poor glycemic control and complications. Dipeptidyl peptidase-4 (DPP-4) inhibitors continue to be an important component of T2DM treatment due to their favorable efficacy and tolerability profile. Given the current scenario, there is a need to revisit the role of DPP-4 inhibitors in T2DM management in Indian patients. This consensus paper aims to provide guidance on the utilization of DPP-4 inhibitors in T2DM management from an Indian perspective.

A consensus group of 100 experts developed recommendations based on an extensive literature review and discussions. The expert group emphasized the importance of timely glycemic control, combination therapy, and targeting the underlying pathophysiology of T2DM. The combinations of DPP-4 inhibitors with metformin and/or sodium-glucose transport protein-2 inhibitors are rationalized in this paper, considering their complementary mechanisms of action.

This paper provides valuable insights for clinicians in optimizing the management of T2DM in the Indian population with the use of DPP-4 inhibitors and proposes an algorithm for selecting DPP-4 inhibitor-based therapies.

## Introduction and background

India is home to the second largest population with diabetes in the world [1]. The South Asian region, including India, has experienced a steady increase in the prevalence of type 2 diabetes mellitus (T2DM), as the resident population is intrinsically predisposed to develop the condition [2]. According to a recent report from the National Non-communicable Disease Monitoring Survey, the prevalence of diabetes in India was recorded at 9.3% in 2018 [3]. T2DM in Indians differs from that in Caucasians in several ways. Indians tend to develop diabetes at a younger age and lower body mass index compared to Caucasians. Additionally, there is a higher risk of developing cardiovascular disease (CVD) among Indians [2].

Despite the availability of newer oral antidiabetic drugs (OADs), there is a concerning trend toward poor glycemic control among people with T2DM in both urban and rural areas of India. The Indian Council of Medical Research-India Diabetes study showed that achieving treatment targets and adopting healthy behaviors remains suboptimal in India. As per this study, approximately 64% of individuals with T2DM do not attain the glycated hemoglobin (HbA1c) target of <7% [4]. According to epidemiological studies, the average HbA1c of Indians living with diabetes is 9%, which is at least 2.0% higher than global standards [5].

Elevated HbA1c levels above 7% have been consistently associated with an increased risk of both micro and macrovascular complications. Individuals with higher baseline values require prompt and intensive treatment to mitigate this risk. Monotherapy often exhibits inadequate efficacy in these individuals, necessitating the initiation of combination therapy at an early stage [6]. A recent community‑based cross‑sectional study reported that most healthcare providers in India do not follow the standard guidelines for treating T2DM and barely satisfy the global standards of diabetes care proposed by various guidelines [7].

With advancements in research, novel therapies to treat diabetes have been introduced, which have received a prominent position in current therapeutic guidelines [8]. Dipeptidyl peptidase-4 (DPP-4) inhibitors represent a safe class of OADs with great clinical experience since their approval in 2006. Evidence suggests that DPP-4 inhibitors are the first-line choice when preference is given to individualized glycemic goals, individualized weight management goals, avoidance of side effects, individual satisfaction, cost, and availability [9].

Given the current scenario, there is a need to revisit its role in the management of T2DM in the Indian context and develop a consensus statement based on current literature and clinical practice-directed expert opinion to guide its optimal usage.

Objectives, scope, and approach in developing the expert consensus recommendations

This expert consensus paper aims to review and discuss the role of DPP-4 inhibitors in T2DM management and provide guidance and recommendations for their effective use in clinical practice settings in India. Based on the identified unmet needs, the scope of the consensus meeting included developing consensus recommendations for optimizing the use of DPP-4 inhibitors for optimal glycemic control and establishing a well-defined treatment algorithm for using DPP-4 inhibitors.

The consensus group was formed by 100 experts representing endocrinologists and consulting physicians across different regions of India. The national coordinator of the panel, in collaboration with other experts, formulated the consensus statements through extensive literature reviews and discussion of available evidence tailored to the Indian context. Every panel member equally contributed significantly to the discussion, with active engagement throughout the consensus development process. The experts engaged in the process of consensus development and algorithm formulation maintained complete independence from any influence from the industry. The writing committee drafted the manuscript, which was subsequently reviewed by all members of the expert panel, guaranteeing a thorough and well-organized document.

## Review

Role of DPP-4 inhibitors in the management of T2DM

Modern guidelines focus on personalized diabetes treatment to enhance patient satisfaction, quality of life, and medication adherence. Shared decision-making helps establish individualized treatment goals, including glycemic control, weight management, comorbidity prevention, and reducing hypoglycemia risk. Personalized diabetes management also addresses factors contributing to morbidity and mortality in T2DM, such as underutilization of evidence-based treatments and limitations in current therapies [10,11].

Individual characteristics encompassing priorities, lifestyle, health behaviors, and comorbidities such as CVD, chronic kidney disease (CKD), and heart failure (HF) influence the choice of antihyperglycemic agents (AHAs). Apart from these, age and weight, motivation, depression, cognition, and social determinants of health also impact glycemic targets. Therapeutic choices depend on personalized HbA1c targets, impact on weight, hypoglycemia risk, side effects, and treatment complexity. Adherence, persistence, cost, and medication availability are essential considerations for personalized glycemic management [12]. Globally, 32% of T2DM patients have CVD, while 68% do not have CVD [13]. For those not having CVD, high atherosclerotic CVD (ASCVD) risk, HF, or CKD, the choice of medication is based on glycemic and weight management goals, side effects, cost, access, and individual preferences [9].

Despite the spotlight on sodium-glucose transport protein-2 (SGLT-2) inhibitors and glucagon-like peptide-1 receptor agonists (GLP-1 RAs) due to their established cardio-renal benefits, the role of DPP-4 inhibitors remains significant due to their favorable efficacy and tolerability profile [14]. They demonstrate intermediate effectiveness in reducing HbA1c levels with minimal risk of hypoglycemia. Additionally, they have neutral effects on body weight and blood pressure, and their cardiorenal safety has been well-established [9]. Furthermore, DPP-4 inhibitors exhibit long-lasting control over blood glucose levels. They can be combined with other OADs and insulin to achieve or maintain desired glucose targets. Moreover, DPP-4 inhibitors can be utilized in patients with T2DM, regardless of the presence of comorbidities such as ASCVD, HF, or CKD [9].

Clinically, there is no substantial difference in HbA1c reduction between SGLT-2 and DPP-4 inhibitors. Multiple studies over the past decade have demonstrated comparable efficacy of both these classes of drugs in lowering HbA1c levels [14]. However, there is a variation in the HbA1c-lowering effect based on the baseline HbA1c values. SGLT-2 inhibitors tend to exhibit more favorable HbA1c reduction when the baseline HbA1c is higher (>8.5%), while DPP-4 inhibitors demonstrate a more favorable effect in individuals with modest baseline HbA1c values (≤8.5%). Although the reasons behind this differential effect are not fully understood, it is known that the HbA1c-lowering ability of SGLT-2 inhibitors depends on the renal threshold for glucose excretion (RTG), and modest baseline HbA1c may not result in further lowering of RTG. Moreover, the glucose-lowering potential of SGLT-2 inhibitors gets compromised with a progressive decline in kidney function, unlike DPP-4 inhibitors [14]. Current evidence also indicates that DPP-4 inhibitors have greater glucose-lowering efficacy in Asians than Caucasians [15].

Different DPP-4 inhibitors became available in the market in 2006. Dissimilarities in the chemical structure of these different DPP-4 inhibitors impact their pharmacokinetic and pharmacodynamic properties. Table 1 summarizes the comparative pharmacology of different DPP-4 inhibitors [16,17].

**Table 1:** Comparative pharmacology of DPP-4 inhibitors. Adopted from [15,16]. DPP-4: dipeptidyl peptidase-4; GLP-1: glucagon-like peptide-1

Parameters	Teneligliptin	Saxagliptin	Linagliptin	Sitagliptin	Vildagliptin
Usual oral daily dose	20 mg	5 mg	5 mg	100 mg	100 mg
Absolute oral bioavailability [%]	63–85	75	30	87	85
Inhibition of plasma DPP-4 activity (24-hour post-dose) [%]	61.8	≥70 with a covalent bond	≥70	≥80	≥80 with a covalent bond
Increase in active GLP-1 levels	-	1.5–2-fold	2–3-fold	2-fold	3-fold
Primary route elimination	Renal and hepatic	Renal and hepatic	Enterohepatic	Renal	Hepatic
Rate of elimination	Slow	Rapid	Slow	Moderate	Rapid
Dose titration in renal impairment	Not required	5 mg: >60 mL/minute; 2.5 mg: ≤60 mL/minute	Not required	100 mg: >60 mL/minute; 50 mg: 30–59 mL/minute; 25 mg: <30 mL/minute	100 mg: >60 mL/minute; 50 mg: ≤60 mL/minute

The effectiveness in achieving glycemic control varies among different DPP-4 inhibitors, with an average reduction in HbA1c ranging from -0.5% to -1.0% when used as monotherapy. Among the DPP-4 inhibitors, vildagliptin, in some reports, has demonstrated superior reductions in glycemic parameters compared to other drugs in the class [18]. Vildagliptin was one of the earliest DPP-4 inhibitors introduced to the market for the management of T2DM following its initial approval in March 2007. Since then, vildagliptin has been approved in over 110 countries, including India, in January 2008. Vildagliptin is a potent, selective, and reversible inhibitor of DPP-4, meaning it specifically targets and inhibits this enzyme. It forms a strong covalent bond with DPP-4, leading to a slow dissociation and providing a longer duration of action [19]. Vildagliptin has a robust evidence base supported by randomized controlled trials and extensive clinical experience over 15 years. It has been studied across the spectrum of T2DM management as part of monotherapy, dual therapy, and triple therapy, including in combination with insulin. It has also been studied in patients with multiple comorbidities, including CKD. Vildagliptin has been shown to have a more favorable effect on reducing glycemic variability than sitagliptin, another DPP-4 inhibitor. Additionally, vildagliptin carries a low risk of hypoglycemia, is weight-neutral, and has been deemed cardiovascular-safe based on meta-analyses conducted in this field [19]. An Indian study showed that once-daily Vildagliptin SR 100 mg is as effective as twice-daily Vildagliptin IR 50 mg. Vildagliptin SR 100 mg provides over 80% DPP-4 inhibition coverage for 24 hours, leading to a significant reduction in glucose levels. Patients need to take fewer pills, improving adherence and diabetes management. The convenient dosing of Vildagliptin SR offers meaningful glycemic control, enhancing overall treatment outcomes for diabetes patients [20]. The VERIFY trial provided compelling evidence that initiating treatment with a combination of vildagliptin and metformin in patients with newly diagnosed T2DM offers superior and long-lasting benefits compared to the conventional approach of initiating treatment with metformin monotherapy. This finding highlights the potential of early combination therapy as an effective and durable approach for managing T2DM [21].

DPP-4 inhibitors have demonstrated favorable safety profile in clinical trials. They do not cause weight gain and are associated with minimal risk of hypoglycemia. DPP-4 inhibitors can also be safely used in more fragile populations such as the elderly and those with renal impairment [22]. Although nasopharyngitis, gastrointestinal symptoms, upper respiratory infections, and headaches are the most frequently reported adverse events, findings have not been consistent across all studies. While these adverse effects were more prevalent compared to placebo in numerous clinical trials, they did not consistently differ significantly from those observed with active comparators. Moreover, the use of DPP-4 inhibitors did not exhibit an elevated risk of hypoglycemia relative to placebo in clinical trials, except when co-administered with sulphonylureas or insulin in certain studies [23]. Studies have indicated increased levels of lipase and amylase, suggestive of pancreatic inflammation, in patients receiving DPP-4 inhibitors compared to control groups. Post-marketing surveillance and case reports have identified isolated cases of pancreatitis associated with the use of DPP-4 inhibitors, although some large-scale studies and meta-analyses have not found significantly increased rates of pancreatitis compared to comparators [23].

With an increased emphasis on the safety and tolerability of drugs, modern research has revealed subtle differences in the pathophysiology, manifestation, and clinical significance of daytime hypoglycemia and nocturnal hypoglycemia. In light of these findings, the earlier glycemic pentad has been expanded to the glycemic hexad. This hexad now includes three efficacy-focused objectives, which include HbA1c, fasting plasma glucose, and postprandial glucose, along with three safety-related targets, which include hypoglycemia, nocturnal hypoglycemia, and glycemic variability [24].

This highlights the need for therapies that can effectively address all five elements. Achieving all aspects may require combination therapy, as monotherapy might not suffice [25]. Continuous glucose monitoring (CGM) has surpassed HbA1c measurements, with time in range (TIR) being a comprehensive indicator of glycemic control. TIR is also predictive of diabetes-related complications. Decreased TIR and increased glucose variability are associated with both microvascular and macrovascular complications associated with diabetes [26]. A retrospective cohort analysis demonstrated that the transition of HbA1c from more than 9% to less than 7.5% reduces the risk of major adverse cardiovascular events (MACE) by 25%. In comparison, glycemic variability is associated with an increased risk of MACE by 51% compared to lower glycemic variability [27].

In this context, vildagliptin has been found to offer superior 24-hour blood glucose control and reduced glycemic variability when compared to sitagliptin. CGM studies have shown that patients with T2DM who were treated with vildagliptin had significantly lower mean 24-hour blood glucose levels, lower mean amplitude of glycemic excursions, and lower highest blood glucose levels after supper than sitagliptin [28]. The area under the curve for blood glucose level ≥180 mg/dL within three hours was significantly lower after breakfast in patients treated with vildagliptin than in patients taking sitagliptin. Furthermore, the levels of B-type natriuretic peptide and plasminogen activator inhibitor-1, which are markers of cardiovascular health, did not show any significant differences between patients taking vildagliptin and those taking sitagliptin. This suggests that both vildagliptin and sitagliptin have a comparable effect on these specific cardiovascular biomarkers. Overall, vildagliptin demonstrated improved 24-hour blood glucose control, reduced glycemic variability, and similar effects on cardiovascular biomarkers compared to sitagliptin in patients with T2DM [28].

According to a comprehensive meta-analysis, including a large study population of over 17,000 patients, with a substantial proportion of patients at an increased cardiovascular risk, vildagliptin has been found to have a cardiovascular and HF safety profile that is comparable to other comparators (placebo and all non-vildagliptin treatments). The analysis indicates that vildagliptin treatment is not associated with an increased risk of MACEs. Additionally, no significant increase in the risk of HF was observed in patients treated with vildagliptin. The findings of this analysis provided reassurance regarding the ongoing use of vildagliptin in the treatment of T2DM. Additionally, these outcomes align with the findings of cardiovascular outcome studies conducted on DPP-4 inhibitors, which collectively support the cardiovascular safety of this class of medications [29].

Effective utilization of DPP4 inhibitor-based combination therapies in T2DM management

Timely achievement of glycemic control is crucial for optimizing clinical outcomes in diabetes. Early and proactive intervention helps maintain patients within the target glycemic range. Guidelines suggest considering combination therapy to achieve treatment goals and recommend initial combination therapy for HbA1c levels 1.5-2.0% above the target [9]. The American Association of Clinical Endocrinology (AACE) 2023 guidelines suggest that early combination therapy with two agents may be needed if the initial HbA1c is >7.5%, and for those with an initial HbA1c of >9% or 1.5% above goal, two or three antihyperglycemic agents should be initiated concomitantly [30]. Initiating combination therapy at an earlier stage has a strong rationale. Early combination therapy achieves treatment goals faster, reducing risks associated with prolonged inadequate glycemic control and reducing microvascular and macrovascular complications and overall mortality [31,32]. Studies showed that even a one-year delay in treatment intensification significantly increases the risk of cardiovascular events [30,33]. However, treatment intensification should align with personalized goals and need not involve strictly sequential addition of therapies [9].

**Figure 1:**
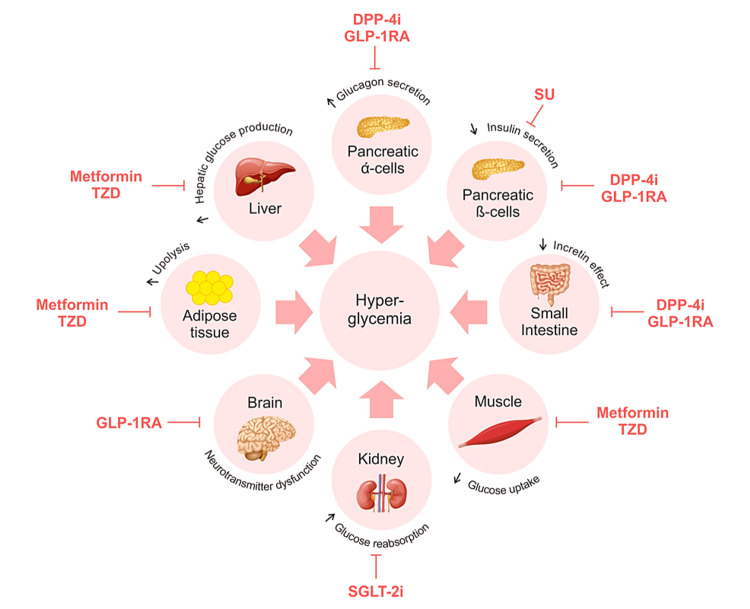
“Ominous octet” and classes of AHAs targeting it. AHA: antihyperglycemic agents; DPP-4i: dipeptidyl peptidase-4 inhibitors; GLP1-RA: GLP-1 receptor agonists; SGLT-2i: sodium-glucose transport protein-2 inhibitors; SU: sulphonylureas; TZD: thiazolidinediones

Targeting the “ominous octet” provides the rationale for the combination therapies. The “ominous octet” refers to the core pathophysiological mechanisms of diabetes. It is crucial to address multiple underlying pathophysiological aspects of T2DM for optimum clinical outcomes of the treatment. Different classes of AHAs act on different components of this “ominous octet” (Figure 1). A pathophysiological approach to treating T2DM involves using combination therapy with medications that target the multiple defects in T2DM [34].

The rationale for DPP-4 inhibitor-based combination therapies

Currently, different DPP-4 inhibitor-based combination therapies are available to treat T2DM. These include dual combinations of DPP-4 inhibitor with metformin or SGLT-2 inhibitors and triple combination of DPP-4 inhibitor with metformin and an SGLT-2 inhibitor. The rationale behind these combinations lies in their complementary mechanisms of action [34]. Metformin reduces hepatic glucose production and enhances insulin sensitivity in the liver and muscles. DPP-4 inhibitors increase GLP-1 levels, stimulating insulin secretion and suppressing glucagon secretion. Metformin also increases GLP-1 levels, and combining it with DPP-4 inhibitors can have a synergistic effect [34,35]. The combination preserves beta-cell function and avoids weight gain, hypoglycemia, and gastrointestinal issues associated with metformin use [36]. The current Research Society for the Study of Diabetes in India (RSSDI)-Endocrine Society of India (ESI) clinical practice guidelines recommend DPP-4 inhibitors as second-line therapy as an add-on to metformin if glucose control targets are not achieved with metformin monotherapy [37].

SGLT-2 inhibitors reduce glucose reabsorption in the kidneys, leading to glycosuria. They can enhance glucagon secretion and increase blood glucose levels, but when combined with DPP-4 inhibitors, this effect is counteracted, resulting in lower blood glucose levels. The combination also improves glycemic variability [38]. SGLT-2 inhibitors also provide additional benefits, such as weight loss and reductions in systolic blood pressure [34,39]. The combination of DPP-4 and SGLT-2 inhibitors may positively affect the kidneys. It has been found that adding a DPP-4 inhibitor to an SGLT-2 inhibitor reduces the incidence of genitourinary infections associated with SGLT-2 inhibitor use [40]. A recently conducted meta-analysis, which examined the cardiovascular and renal benefits of combining SGLT-2 inhibitors with DPP-4 inhibitors, revealed that the positive cardiovascular and renal effects of SGLT-2 inhibitors were consistent and similar compared to the placebo group, irrespective of background DPP-4 inhibitor therapy [41].

**Figure 2:**
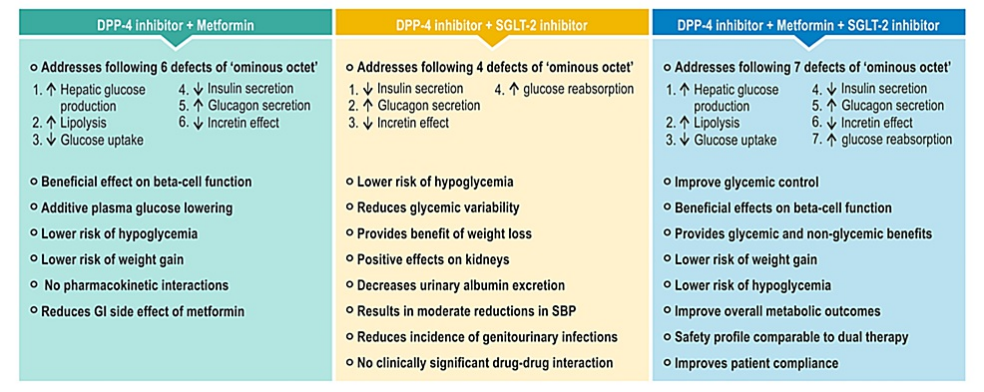
Class-specific complementary and synergistic benefits of DPP-4 inhibitor-based combination therapies. DPP-4: dipeptidyl peptidase-4; SGLT-2: sodium-glucose transport protein-2

Triple therapy comprising an SGLT-2 inhibitor, a DPP-4 inhibitor, and metformin in managing T2DM has demonstrated notable enhancements in blood glucose regulation, body weight management, and blood pressure compared to dual therapy. The overall safety profile of this triple therapy remains generally comparable to dual therapy [42]. Along with the benefits provided by individual drugs, the triple combination has been shown to improve the overall metabolic profile of patients. It also enhances patient compliance, making it a valuable choice for individuals requiring diabetes management [34]. RSSDI-ESI clinical practice guidelines also recommend triple therapy with metformin, a DPP-4 inhibitor. and an SGLT-2 inhibitor if glycemic targets are not achieved with dual therapy [37]. Figure 2 summarizes the class-specific complementary and synergistic benefits of DPP-4 inhibitor-based combination therapies.

Consensus on the use of DPP4 inhibitor-based therapies in T2DM management

Consensus Statements

Consensus 1: Approximately 30-40% of patients with T2DM on medications fail to achieve their individualized HbA1c target.

Consensus 2: The average baseline HbA1c levels in patients presenting with T2DM typically range from 8.1% to 9%.

Consensus 3: Key patient characteristics, including age, HbA1c level, weight, and the presence of comorbidities such as ASCVD, CKD, and HF, are crucial factors for selecting appropriate AHAs in the management of T2DM.

Consensus 4: Around 20-30% of T2DM patients have established ASCVD.

Consensus 5: Efficacy and cardio-renal safety are the primary factors that determine the choice of AHAs for T2DM patients without established ASCVD, HF, or CKD.

Consensus 6: Early initiation of combination therapy in managing T2DM is associated with early and greater reductions in HbA1c, durable glycemic efficacy, improvement in beta-cell function over time, and delayed clinical complications by addressing multiple pathophysiological defects.

Consensus 7: In the natural history of T2DM, DPP-4 inhibitor-based therapy is considered most suitable for newly diagnosed patients without comorbidities, as well as for patients without established ASCVD, HF, or CKD, where the primary treatment goals involve achieving optimal glycemic control, managing body weight, and minimizing the risk of hypoglycemia.

Consensus 8: Vildagliptin is one of the preferred DPP-4 inhibitors due to its greater efficacy, established durability, and better effect on glycemic variability.

Consensus 9: Vildagliptin SR in combination with metformin SR fixed-dose combination may be preferred in newly diagnosed T2DM patients with HbA1c >7.5% and in patients who are uncontrolled on metformin with HbA1c >7.5%. This regimen may also be preferred to reduce the pill burden in patients with polypharmacy.

Consensus 10: DPP-4 inhibitors are considered safe/neutral in terms of cardiovascular outcomes, including HF (except saxagliptin), and can be a useful add-on to existing AHAs, including insulin.

Consensus Algorithm for Selecting DPP-4 Inhibitor-Based Therapies in Indian Patients With T2DM

**Figure 3:**
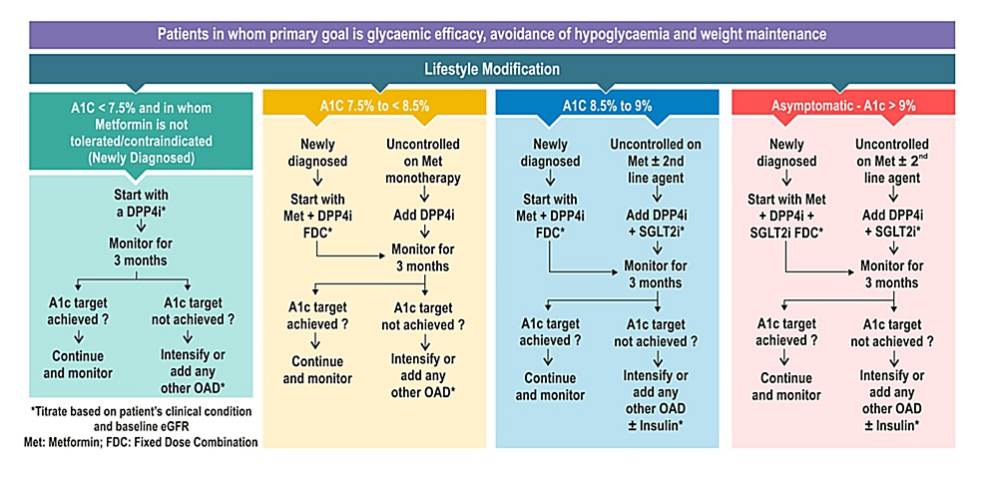
Proposed algorithm for DPP-4 inhibitor-based therapies in the management of T2DM without established ASCVD, HF, or CKD. ASCVD: atherosclerotic cardiovascular disease; CKD: chronic kidney disease; DPP-4i: dipeptidyl peptidase-4 inhibitors; FDC: fixed-dose combination; HF: heart failure; Met: metformin; OAD: oral antidiabetic drug; SGLT-2i: sodium-glucose transport protein-2 inhibitors; T2DM: type 2 diabetes mellitus

As mentioned earlier in the article, DPP-4 inhibitors have been utilized to treat patients with T2DM, regardless of the presence of comorbidities such as ASCVD, HF, or CKD. However, with advancements in diabetes treatment, more effective therapies have emerged for T2DM patients with these comorbidities, which has led to repositioning the role of DPP-4 inhibitors, primarily in T2DM patients without these conditions. It is important to acknowledge that the majority of T2DM patients fall within the category of those without established ASCVD, suggesting that optimizing DPP-4 inhibitor-based treatments will greatly benefit this significant group of patients. As a response to this, the consensus group has proposed an algorithm to guide the selection of DPP-4 inhibitor-based therapies along with lifestyle modifications in managing T2DM in Indian patients without established ASCVD, HF, or CKD, where the primary goal is glycemic efficacy, weight management, and avoidance of hypoglycemia (Figure 3).

This algorithm is developed based on the current evidence-based clinical practice of Indian experts involved in consensus development and reference algorithm by the AACE 2020 for comprehensive management of T2DM [43] and recently updated AACE 2023 guidelines [30].

For patients with newly diagnosed T2DM with mild hyperglycemia (HbA1c <7.5%) and in whom metformin is not tolerated or contraindicated, lifestyle therapy plus antihyperglycemic monotherapy with a DPP-4 inhibitor may be initiated with a dose titrated based on the patient’s clinical condition and baseline eGFR. In patients who do not reach their glycemic target on DPP-4 inhibitor monotherapy after three months, it is suggested that the treatment be intensified with any other OAD.

T2DM patients who present with an HbA1c ≥7.5% to <8.5% at diagnosis may be started on metformin + DPP-4 inhibitor fixed-dose (single-pill) combination therapy. An add-on therapy with a DPP-4 inhibitor as a single pill may be considered for patients uncontrolled on metformin monotherapy with HbA1c ≥7.5% to <8.5%. After three months, it is suggested that the treatment be intensified or that any other OAD be added in patients who do not reach their glycemic target on metformin and DPP-4 inhibitor combination therapy. Newly diagnosed T2DM patients with HbA1c between ≥8.5% and <9% may be initiated with metformin + DPP-4 inhibitor single-pill combination therapy. In T2DM patients uncontrolled on metformin with or without a second-line agent and DPP-4 inhibitor + SGLT-2 inhibitor combination therapy may be considered. In patients not achieving glycemic targets, it is suggested to intensify the treatment or add any other OAD with or without insulin therapy after three months.

In newly diagnosed asymptomatic T2DM patients with HbA1c ≥9%, treatment may be started with a triple-drug single-pill combination of metformin, DPP-4 inhibitor, and SGLT-2 inhibitor. The addition of DPP-4 inhibitor and SGLT-2 inhibitor fixed-dose combination therapy may be considered in asymptomatic T2DM patients who are uncontrolled on metformin with or without a second-line agent and HbA1c ≥9%. It is suggested to intensify the treatment or add any other OAD with or without insulin therapy in patients not achieving glycemic targets after three months. Throughout the algorithm, titrating the dose of OADs, including DPP-4 inhibitors, is suggested based on the patient’s clinical condition and baseline eGFR.

It has to be noted that if a patient, during the course of DPP4 inhibitor-based therapy, is diagnosed with ASCVD, HF, or CKD, it is recommended, in accordance with established guidelines, to consider the initiation of treatment with an SGLT-2 inhibitor or a GLP-1 RA with proven cardiorenal benefits, provided that the patient is not already receiving these agents.

## Conclusions

This paper summarizes the role of DPP-4 inhibitors in managing T2DM in India. It highlights the importance of early combination therapy to control blood sugar levels effectively. Despite efforts, many T2DM patients in India struggle to reach target HbA1c levels. Early combination therapy can help achieve better and lasting control over time, reducing complications. Individualized treatment, considering patients’ unique characteristics and health conditions such as CVD, kidney disease, and HF, is crucial. DPP-4 inhibitors, especially vildagliptin, are recommended for T2DM patients without these conditions due to their effectiveness and safety. These expert recommendations aim to improve diabetes management in India by optimizing the use of DPP-4 inhibitors, ultimately leading to better outcomes for T2DM patients.

## References

[1] Das AK, Mohan V, Joshi S (2021). IMPACT India: a novel approach for optimum diabetes care. J Diabetol.

[2] Staimez LR, Deepa M, Ali MK, Mohan V, Hanson RL, Narayan KM (2019). Tale of two Indians: heterogeneity in type 2 diabetes pathophysiology. Diabetes Metab Res Rev.

[3] Mathur P, Leburu S, Kulothungan V (2022). Prevalence, awareness, treatment and control of diabetes in India from the countrywide National NCD Monitoring Survey. Front Public Health.

[4] Anjana RM, Unnikrishnan R, Deepa M (2022). Achievement of guideline recommended diabetes treatment targets and health habits in people with self-reported diabetes in India (ICMR-INDIAB-13): a national cross-sectional study. Lancet Diabetes Endocrinol.

[5] Joshi SR (2015). Diabetes care in India. Ann Glob Health.

[6] Stratton IM, Adler AI, Neil HA (2000). Association of glycaemia with macrovascular and microvascular complications of type 2 diabetes (UKPDS 35): prospective observational study. BMJ.

[7] Dixit JV, Kulkarni RS, Badgujar SY (2021). Diabetes care in India: a descriptive study. Indian J Endocrinol Metab.

[8] Florentin M, Kostapanos MS, Papazafiropoulou AK (2022). Role of dipeptidyl peptidase 4 inhibitors in the new era of antidiabetic treatment. World J Diabetes.

[9] ElSayed NA, Aleppo G, Aroda VR (2023). 9. Pharmacologic approaches to glycemic treatment: standards of care in diabetes-2023. Diabetes Care.

[10] Williams DM, Jones H, Stephens JW (2022). Personalized type 2 diabetes management: an update on recent advances and recommendations. Diabetes Metab Syndr Obes.

[11] Grant RW, Wexler DJ (2012). Personalized medicine in type 2 diabetes: what does the future hold?. Diabetes Manag (Lond).

[12] ElSayed NA, Aleppo G, Aroda VR (2023). 4. Comprehensive medical evaluation and assessment of comorbidities: standards of care in diabetes-2023. Diabetes Care.

[13] Einarson TR, Acs A, Ludwig C, Panton UH (2018). Prevalence of cardiovascular disease in type 2 diabetes: a systematic literature review of scientific evidence from across the world in 2007-2017. Cardiovasc Diabetol.

[14] Singh AK, Singh R (2022). Relook at DPP-4 inhibitors in the era of SGLT-2 inhibitors. World J Diabetes.

[15] Cai X, Han X, Luo Y, Ji L (2015). Efficacy of dipeptidyl-peptidase-4 inhibitors and impact on β-cell function in Asian and Caucasian type 2 diabetes mellitus patients: a meta-analysis. J Diabetes.

[16] Golightly LK, Drayna CC, McDermott MT (2012). Comparative clinical pharmacokinetics of dipeptidyl peptidase-4 inhibitors. Clin Pharmacokinet.

[17] Maladkar M, Sankar S, Kamat K (2016). Teneligliptin: heralding change in type 2 diabetes. J Diabetes Mellitus.

[18] Subrahmanyan NA, Koshy RM, Jacob K, Pappachan JM (2021). Efficacy and cardiovascular safety of DPP-4 inhibitors. Curr Drug Saf.

[19] Keating GM (2014). Vildagliptin: a review of its use in type 2 diabetes mellitus. Drugs.

[20] Joshi HR, Joshi N, Warrier S (2022). A comparative pharmacodynamic and pharmacokinetic study of Vildagliptin SR 100 mg tablet in normal healthy adult male subjects. J Drug Delivery Ther.

[21] Matthews DR, Paldánius PM, Proot P, Chiang Y, Stumvoll M, Del Prato S (2019). Glycaemic durability of an early combination therapy with vildagliptin and metformin versus sequential metformin monotherapy in newly diagnosed type 2 diabetes (VERIFY): a 5-year, multicentre, randomised, double-blind trial. Lancet.

[22] Scheen AJ (2015). Safety of dipeptidyl peptidase-4 inhibitors for treating type 2 diabetes. Expert Opin Drug Saf.

[23] Filippatos TD, Athyros VG, Elisaf MS (2014). The pharmacokinetic considerations and adverse effects of DPP-4 inhibitors [corrected]. Expert Opin Drug Metab Toxicol.

[24] Kalra S (2018). The glycaemic sixer [glycaemic hexad]. J Pak Med Assoc.

[25] Forum GP (2017). Glycemic pentad. J Assoc Physicians India.

[26] Yoo JH, Kim JH (2020). Time in range from continuous glucose monitoring: a novel metric for glycemic control. Diabetes Metab J.

[27] Whyte MB, Joy M, Hinton W (2022). Early and ongoing stable glycaemic control is associated with a reduction in major adverse cardiovascular events in people with type 2 diabetes: a primary care cohort study. Diabetes Obes Metab.

[28] Sakamoto M, Nishimura R, Irako T, Tsujino D, Ando K, Utsunomiya K (2012). Comparison of vildagliptin twice daily vs. sitagliptin once daily using continuous glucose monitoring (CGM): crossover pilot study (J-VICTORIA study). Cardiovasc Diabetol.

[29] McInnes G, Evans M, Del Prato S (2015). Cardiovascular and heart failure safety profile of vildagliptin: a meta-analysis of 17 000 patients. Diabetes Obes Metab.

[30] Samson SL, Vellanki P, Blonde L (2023). American Association of Clinical Endocrinology Consensus Statement: comprehensive type 2 diabetes management algorithm - 2023 update. Endocr Pract.

[31] Khunti K, Millar-Jones D (2017). Clinical inertia to insulin initiation and intensification in the UK: a focused literature review. Prim Care Diabetes.

[32] Bailey CJ, Del Prato S, Eddy D, Zinman B (2005). Earlier intervention in type 2 diabetes: the case for achieving early and sustained glycaemic control. Int J Clin Pract.

[33] Paul SK, Klein K, Thorsted BL, Wolden ML, Khunti K (2015). Delay in treatment intensification increases the risks of cardiovascular events in patients with type 2 diabetes. Cardiovasc Diabetol.

[34] Chadha M, Das AK, Deb P (2022). Expert opinion: optimum clinical approach to combination-use of SGLT2i + DPP4i in the Indian diabetes setting. Diabetes Ther.

[35] Ahrén B (2008). Novel combination treatment of type 2 diabetes DPP-4 inhibition + metformin. Vasc Health Risk Manag.

[36] Halimi S, Schweizer A, Minic B, Foley J, Dejager S (2008). Combination treatment in the management of type 2 diabetes: focus on vildagliptin and metformin as a single tablet. Vasc Health Risk Manag.

[37] Chawla R, Madhu SV, Makkar BM, Ghosh S, Saboo B, Kalra S (2020). RSSDI-ESI clinical practice recommendations for the management of type 2 diabetes mellitus 2020. Indian J Endocrinol Metab.

[38] Cho KY, Nomoto H, Nakamura A (2021). Improved time in range and postprandial hyperglycemia with canagliflozin in combination with teneligliptin: secondary analyses of the CALMER study. J Diabetes Investig.

[39] Sharma MD (2015). Potential for combination of dipeptidyl peptidase-4 inhibitors and sodium-glucose co-transporter-2 inhibitors for the treatment of type 2 diabetes. Diabetes Obes Metab.

[40] Scheen AJ (2016). DPP-4 inhibitor plus SGLT-2 inhibitor as combination therapy for type 2 diabetes: from rationale to clinical aspects. Expert Opin Drug Metab Toxicol.

[41] Singh AK, Singh A, Singh R (2023). Cardiovascular and renal outcomes with sodium-glucose cotransporter-2 inhibitors and dipeptidyl peptidase-4 inhibitors combination therapy: a meta-analysis of randomized cardiovascular outcome trials. Endocr Pract.

[42] Li M, Wang S, Wang X (2023). Efficacy and safety of triple therapy with SGLT-2 inhibitor, DPP-4 inhibitor, and metformin in type 2 diabetes: a meta-analysis. Altern Ther Health Med.

[43] Garber AJ, Handelsman Y, Grunberger G (2020). Consensus statement by the American Association of Clinical Endocrinologists and American College of Endocrinology on the comprehensive type 2 diabetes management algorithm - 2020 executive summary. Endocr Pract.

